# State Medical Society of Iowa

**Published:** 1859-09

**Authors:** 


STATE MEDICAL SOCIETY OF IOWA.—This body met at Davenport, on the seventh, eighth, and ninth of June, the attendance being larger than on any previous occasion, about fifty members, representing fifteen counties, being present. The retiring President, Dr. John H. Rauch, who has been called to fill the Chair of Materia Medica in a Chicago medical college, delivered a very eloquent address, a copy of which was requested for publication. A number of important papers were read, among which we may mention the elaborate report of Dr. Rauch, on the Topography, Natural History, and Climate of Iowa; and an essay on the Medical Properties of the Chlorate of Potassa, by Dr. E. J. Fountain. The following gentlemen were elected officers of the Society for the ensuing year:—*President*, Dr. E. S. Barrows; *Vice-President*, Dr. G. Reeder; *Recording Secretary*, Dr. A. Phillips; *Corresponding Secretary*, Dr. Oliver George; *Treasurer*, Dr. M. B. Cochran; *Censors*, Drs. Fountain, McGugin, Lewis, McClure, and Dimmitt. The meeting was exceedingly pleasant and harmonious, and the Society adjourned to meet at Dubuque on the second Wednesday of May, 1860.

